# Effects of *Pichia manshurica* yeast supplementation on ruminal fermentation, nutrient degradability, and greenhouse gas emissions in aflatoxin B1 contaminated diets

**DOI:** 10.1007/s11250-024-04184-8

**Published:** 2024-10-30

**Authors:** Yosra Soltan, Amr Morsy, Mahmoud Elazab, Amr E. El-Nile, Nesrein Hashem, Mohamed Sultan, Younis Hamad, Gomaa Abo El Lail, Sohila Abo-Sherif, Nassra Dabour, Ehab Kheadr, Elsayed Hafez, Sobhy Sallam

**Affiliations:** 1 Faculty of Agriculture, Alexandria University, Alexandria, Egypt; 2 Arid Lands Cultivation Research Institute, City of Scientific Research and Technological Applications, Alexandria, Egypt

**Keywords:** AFB1, Antimicrobial metabolites, Carbone dioxide, Fiber degradability, Methane, Protozoa, Yeast

## Abstract

Yeast feed additives present a natural approach for mitigating ruminal greenhouse gases (GHG) in an environmentally sustainable manner. This study aimed to isolate yeast strains from ruminal fluids capable of reducing GHG from Aflatoxin (AFB1) contaminated diets. Two isolates of *Pichia manchuria* (FFNLYFC1 and FFNLYFC2) were isolated and identified from the ruminal contents of dairy *Zaraibi* goats. An in vitro gas production assay was conducted to evaluate the impact of the yeast supplementations on a basal diet contaminated with AFB1 or not. The treatments were control (-AFB1; basal diet without supplements), control with AFB1 contamination (+ AFB1; basal diet containing 20 ppb AFB1), and yeast-supplemented diets (basal diet supplemented with *Saccharomyces cerevisiae*, and three treatments of *P. manchuria* [FFNLYFC1, FFNLYFC2, and their mixture at 1:1 ratio (Mix)]. High biological components were detected in abundance of both FFNLYFC1, FFNLYFC2 filtrates (e.g., diisooctyl phthalate). The Mix and FFNLYFC2 of *P. manchuria* reduced (*P* < 0.05) methane by 23.5 and 20.8%, respectively, while only Mix inhibited carbon dioxide by 44% compared to the + AFB1 diet. All yeast diets improved (*P* < 0.05) ammonia concentration, total protozoal and *Entodinium spp.* counts compared to + AFB1 diet. The Mix exhibited higher (*P* < 0.05) values of ruminal degraded cellulose, total short-chain fatty acids, acetate and propionate compared to the individual isolates diets. The results suggest synergistic interactions among *P. manshurica* isolates, leading to enhanced ruminal fermentation and reduced GHG emissions while alleviating the adverse effects of AFB1. Therefore, we recommended the Mix of *P. Manchuria* as a novel feed additive to ruminant diets.

## Introduction

The simultaneous increase in greenhouse gases (GHG) is a pressing environmental concern, exacerbating the challenges of global warming and its various impacts (Belanche et al. 2023). Among these impacts, the interplay between global warming and the prevalence of mycotoxins is becoming increasingly significant (Soltan et al. 2022). Aflatoxins, which are toxic metabolites produced by certain species of fungi, are influenced by environmental factors such as temperature and humidity. As global temperatures rise, the geographic distribution and concentration of mycotoxigenic fungi are expected to shift, potentially leading to higher levels of aflatoxins in various regions (Medina et al. 2017; Soltan et al. 2022). Consequently, it seems that strategies targeting both GHG mitigation and mycotoxins are likely to be more effective.

Aflatoxin B1 (AFB1) stands out as the most toxic among mycotoxins, categorized in group 1 due to its cancer-causing properties (Intanoo et al. 2018). When ruminants consume AFB1-contaminated diets, AFB1 can pass through the rumen wall and the small intestine upon ingestion. It undergoes biotransformation into aflatoxin M1 (AFM1) in the liver and and is later excreted biological fluids (e.g., milk) and tissues (Medina et al. 2017). Moreover, AFB1 was found to decrease the ruminal organic matter and fiber degradability, and consequently adversely affects the animal production (milk or meat). Thus, with GHG emissions continuing to rise, the complex relationship between global warming and AFB1 contamination becomes increasingly important [Soltan et al. 2022).

Various strategies were employed to decrease methane (CH_4_) emission or the contamination of animal feeds by AFB1. Methane mitigation strategies, particularly those focused on enhancing the fiber degradability or reduce CH_4_ per unit of degraded fiber, while concurrently enhancing overall animal health and animal production, emerge as the most promising methods. Similarly, biological decontamination of AFB1 has garnered significance and demonstrates a considerable promise as an approach. Its benefits over chemical or physical decontamination methods include economic advantages, enhanced safety, and environmentally friendly characteristics (Intanoo et al. 2018). Several exogenous (e.g. *S. cerevisiae*) and endogenous (e.g. *Kluyveromyces marxianus*, and *Pichia kudriavzevii*) yeast strains have been shown to reduce AFB1 levels in liquid media either by adsorption and/or degradation mechanisms (Intanoo et al. 2018). Moreover yeasts exhibit unique properties that make them well-suited for use as a dietary supplement in ruminant diets. Notably, S. cerevisiae (the most common yeast) has been shown to enhance rumen fermentation processes, as it has the potential to elevate rumen pH through an increase in short chain fatty acids (SCFAs) concentration, alongside a reduction in lactic acid concentration (Sousa et al. 2018). Furthermore, it was reported that the live S. cerevisiae may utilize ruminal hydrogen to enhance the activity of acetogenesis and acetate production; however the literature did not confirm the ruminal CH_4_ reduction effects of S. cerevisiae supplementation (Palangi and Lackner 2022). Moreover S. cerevisiae has no direct effect on fiber degradability S. cerevisiae as it does not release fiber-degrading enzymes (e.g., cellulase or xylanase) (Palangi and Lackner 2022).

It was hypothesized that yeast strains isolated from ruminal fluids that possess both fiber-degrading ability and the capacity to mitigate the effects of AFB1 contamination have the potential to be applied as natural feed additives to sustain animal production under increasing global warming conditions. Therefore, the objective of this experiment is to (1) identify and isolate yeast strains from ruminal fluids of dairy goats fed on high fiber diets for their ability to degrade the dietary fiber, (2) Test the potential of the isolated yeast to degrade the dietary organic matter and fiber contents under the ruminal conditions. (3) To assess the synergetic supplementation effects of these yeast isolate thus these yeasts were evaluated individually or in a mixture.

## Materials and methods

This study was carried out at the Nanotechnology and Greenhouse Gases Laboratory, Faculty of Agriculture at Alexandria University.

### Isolation of the ruminal endogenous fiber degraded yeasts

Approximately 50 ml of ruminal fluid was collected from each of the forty-four Zaraibi goats (27 ± 8.5 kg SE) using an oesophageal probe, three hours after the morning feeding. Yeats isolation was performed using ten ml of the ruminal sample was diluted with 90 ml of sterilized peptone water (0.1%). The diluted sample was subsequently serially diluted by a factor of 10 using peptone water. Appropriate dilutions were then plated onto yeast extract peptone dextrose agar medium (YPD) supplemented with 0.03 g/liter chloramphenicol according to Spencer and Spencer (1997). The plates were then incubated (FISHER SCIENTIFIC/Ottawa, ON, Canada) at 39 °C for 48 h. After incubation, three to five distinct colonies from each plate were chosen and streaked at least five times on the same medium for purification purposes (Spencer and Spencer 1997). All the reagents and mediums that were used in this step were provided form Hi-Media Laboratories Pvt., Maharashtra of India.

The activities of the endoglucanase (cellulase) and xylanase secreted were tested as described by Ranilla et al. (2008) and Soltan et al. (2013) for all the isolates of the endogenous yeasts by determining the reducing sugar release rate upon the hydrolysis of the carboxymethyl cellulose (CMC ≥ 99%; CAS Number 9004-32-4 Sigma- Aldrich, USA) and xylan (≥ 99%; CAS Number: 58–86,6 Sigma/Aldrich, USA). The substrates of CNC and xylan were prepared at 10 g/l, while the solutions of yeast filtrates were prepared by dissolving 0.5 mg dried yeast/ml in 1 mM sodium phosphate buffer. In triplicate, the substrate solutions (0.5 ml) were incubated at 39 °C and a pH of 6.8 with 0.5 ml sodium phosphate buffer and 80 µl of the yeast filtrate solutions for 15 and 5 min for endoglucanase and xylanase analysis, respectively. Standard solutions of the released sugars were prepared using gradually increasing concentrations (from 0 to 2 g/L) of pure glucose [D(+) glucose monohydrate, Sigma-Aldrich, USA] and xylose [D(+) xylose; Sigma-Aldrich, USA] dissolved in sodium phosphate buffer. For both enzymes, the absorbance was read at 540 nm using a spectrophotometer (Alpha-1101 model; Labnics, California, USA) against glucose or xylose standards. From several isolates, we identified just two yeast isolates, namely FFNLYFC1 and FFNLYFC2 that can degrade both CMC and xylan from two different animals.

### Molecular identification of yeasts isolates

DNA extraction from pure yeast isolates cultures using the primers ITS1 and ITS4 (Ferrer et al. 2001), which cover the region of the rDNA containing the 5.8 S rRNA gene and the two ribosomal internal transcribed spacer (ITS1 and ITS2) regions (referred to here as the 5.8 S-ITS region), were employed to amplify the DNA extracted from the yeast cells. The amplification process took place in a programmable heating incubator (MJ Research Inc., Madison, WI, USA). The polymerase chain reaction conditions were similar to those outlined by Esteve-Zarzoso et al. (1999). The PCR mixture had a final volume of 100 µL, containing 2 µL of template DNA (approximately 50 ng), 1 µL of each primer at a concentration of 0.5 µmol/L, 0.25 mmol/L of each deoxynucleoside triphosphate, 2.5 mmol/L MgCl₂, 10 µL of 10× PCR buffer (Invitrogen), and 1.25 U of Taq polymerase. The PCR was conducted for 35 cycles under the following conditions: denaturation at 94 °C for 1 min, annealing at 56 °C for 2 min, and extension at 72 °C for 2 min. Initial denaturation was carried out at 94 °C for 3 min, followed by a final extension at 72 °C for 10 min. The PCR products were separated by electrophoresis on a 0.8% agarose gel (Invitrogen Life Technologies, Carlsbad, CA, USA) in 1X TAE buffer containing 40 mM Tris-acetate and 1 mM EDTA. After staining with ethidium bromide, the bands were visualized using UV photography (Gel Doc, BioRad, Mississauga, Canada).

Sequencing of the 26 S rDNA gene at the D1/D2 region and the internal transcribed spacer (ITS), which is divided into ITS1 and ITS2 by the conserved 5.8 S rDNA gene, are currently employed as molecular methods for yeast identification (Merchánet al. 2020). In this study, the sequencing of ITS/5.8 S rDNA was amplified from tested isolated and compared with similar data in Genebank. The molecular analysis showed that all the isolates belong to different species of *Pichia*. The FFNLYFC1 and FFNLYFC2 were genetically identified as *Pichia manshurica* with identity percent of 82, and 84, respectively. All sequence data were submitted to the GenBank, and the accession numbers are provided in Table 1.

**Table 1:** Molecular identification of yeast isolates

Isolates	Source	Identification	GenBank Accession number	Identity (%)
FFNLYFC1	Rumen of Egyptian Zarbi goat	*Pichia manshurica*	OP600624	82%
FFNLYFC2	Rumen of Egyptian Zarbi goat	*Pichia manshurica*	OP600623	84%

### Gas chromatography/mass spectrometry for FFNLYFC1 and FFNLYFC2 filtrate

For a comprehensive analysis of bioactive components in FFNLYFC1 and FFNLYFC2 yeast filtrate, 10 g of each isolate powder was extracted in 100 ml of ethanol solution (70%) at 40 °C for 72 h. The resulting extract was filtered and the collected filtrate was evaporated at 45 °C until complete dryness. The residues obtained were stored at -20 °C until further use in a laboratory freezer (FNU-MT301B, Fresh, 10th of Ramadan City, Egypt). The chemical constituents of the yeast filtrate ethanolic extract were determined using gas chromatography/mass spectrometry (GC; Thermo Scientific TRACE- 1300 series), equipped with a fused silica DB-5 capillary column (30 m × 0.32 mm id, 0.25 μm film thickness), and coupled to a Triple Quadrupole Mass (TSQ 8000 Evo). The separation conditions were as described by Soltan et al. (2021a) and all the used reagents at this step were from Sigma Chemie GmbH, Steinheim in Germany.

### The in vitro experiment

#### The basal diet and treatments

A basal diet formulated to meet the nutrient requirements of dairy goats (NRC, 2007) recommendations was used as a substrate for the in vitro evaluation of the dose-response effects of the prepared products compared to monensin (a dietary antibiotic that is widely used to reduce the GHG from ruminants). The diet (Table 2) was chemically analyzed according to the AOAC (2006). The basal diet, whether contaminated or not with a final concentration of AFB1 at 20 ppb (sourced from *Aspergillus flavus*, 98% purity, Sigma Chemical Co, Louis, Missouri, USA), was supplemented with the prepared yeasts using the in vitro gas production assay. The total aflatoxins (AFs) present in the basal diet were extracted and purified using VICAM immunoaffinity columns (VI-CAM Aflatest, Milford, MA, USA) following the method described by Hafez et al. (2021). Quantification was performed using the VICAM fluorometry method (VICAM Series 4EX Fluorometer, Milford, MA, USA) according to the manufacturer’s instructions. The final concentrations of AFs in the basal substrate diet contaminated with AFB1 were determined to be 31 ppb. These concentrations exceed the Egyptian maximum permissible levels for AFs and AFB1 (20 ppb and 10 ppb, respectively) in dairy animal feeds (FAO 2004).

**Table 2:** Ingredients and chemical composition based on dry matter (DM) of the experimental basal substrate in the in vitro experiment

Item	Basal diet
Ingredients (%)	
Wheat straw	5.0
Ground maize	10.0
Wheat bran	8.5
Soybean meal	4.0
Barley	21.2
Brocken rice	4.5
Clover hay (*Trifolium alexandrinum)*	45.0
Lime stone	0.8
Sodium chloride	0.5
Vitamins and minerals mixture	0.5
Chemical composition (%)	
Organic matter	91.1
Crude protein	16.5
Neutral detergent fiber	47.0
Acid detergent fiber	12.7
Acid detergent lignin	13.8
Cellulose	11.7
Hemicellulose	20.5
Ether extract	1.58

The indigenous yeasts were supplemented individually or in a mixture as feed additives for the basal substrate diet compared to *Saccharomyces cerevisiae* the most famous exogenous yeast used as a feed supplement for ruminants. The treatments included a control group (basal diet without any supplements), AFB1 diet (basal diet supplements with 20ppb AFB1), FFNLYFC1, FFNLYFC2, Mix of FFNLYFC1 and FFNLYFC2 in a ratio of (1:1). All the yeasts were supplemented at (1.4 × 108 CFU/ g DM).

#### The in vitro gas production (GP) procedure

All treatments were assessed using the semi-automatic GP system (Soltan et al. 2022). Ruminal contents were gathered separately from three fasted slaughtered buffalo calves were obtained from the slaughterhouse affiliated with the Faculty of Agriculture, Alexandria University. These calves were fed *ad libitum* on *Trifolium alexandrinum* hay and rice straw, supplemented with a commercial concentrate mixture containing 12% crude protein. The ruminal contents were transferred into pre-warmed thermo-containers (39 °C) and promptly transported to the laboratory. Upon arrival, the ruminal (solid and liquid) contents collected from the three claves were combined (1:1:1), mixed for 10 s, strained through three layers of cheesecloth, and kept in a water bath (39 °C) under anaerobic conditions (CO_2_ flushing) to obtain the inoculum for the in vitro incubation.

Six incubation bottles were prepared for each treatment; the procedure involved incubating blank bottles, which contained inoculum and buffer solution to adjust the net GP from the inoculum. Experimental diets (500 mg) were precisely weighed into numbered Ankom filter bags (ANKOM Technology Corporation, Fairport, USA), which had been pre-rinsed and pre-weighed with acetone. These bags were then placed into incubation GP bottles, with or without additives, and incubated with 30 ml of buffer incubation medium [Menke’s buffered medium which contains macro-minerals (e.g., ammonium bicarbonate, sodium bicarbonate, disodium phosphate, potassium dihydrogen phosphate, and magnesium sulfate heptahydrate), micro-minerals solution, and resazurin all solved in distilled water)] as described by Kholif et al. (2023) and 15 ml of ruminal inoculum, while maintaining a headspace of 75 ml (Soltan et al. 2022). The bottles were sealed with 20 mm butyl rubber stoppers and aluminum seals, manually shaken, and incubated at 39 °C in a forced air oven (OCT 100, Chem-Tech Company, kafr El sheikh, Egypt) for 24 h.

The gas pressure in the headspace was measured at 4, 8, 12, and 24 h from the start of incubation using a Pressure Logger (model PSI-V2, SmartLab, Cairo, Egypt). Gas production volume (GP) was calculated using the equation: GP (ml) = 6.5465 × *p* − 0.9573 (*n* = 600; R^2^ = 0.99), where GP represents gas volume in milliliters, and p denotes measured pressure in psi.

### Greenhouse gas production

Methane and CO_2_ concentrations were measured using a laser gas Pro detector (Gas-Pro-IR-W368334/01–001, Crowcon Detection Instruments Ltd, Abingdon, United Kingdom) (Kholif et al. 2023). Before the gases determination, the detector was calibrated promptly in accordance with the LRQA-validation ISO9001 quality manual, utilizing a standard calibration gas mixture prepared following BS EN ISO 6145-1-2008.

### Ruminal nutrient degradability

All bags containing the non-degraded substances were retained for the determination of ruminal nutrient degradability. These bags were promptly removed from the bottles and placed on ice to stop the microbial fermentation process. Subsequently, the bags were sequentially treated for 1 h at 90 °C in a semi-automatic fiber analyzer (FA-12, fiber analyzer unit, SmartLab, Cairo, Egypt) with neutral detergent solution, washed, dried, and weighed. They were then subjected to treatment with acid detergent solution for another hour, washed, dried, and weighed. H_2_SO_4_ (72%) treatment was applied to all bags to determine acid detergent lignin (ADL) contents, and finally, they were incinerated to ash. The difference between the incubated organic matter (OM) amounts and those remaining non-degraded was considered to be the truly degraded organic matter (TDOM) following the method outlined by Blümmel et al. (1997). Similarly, the differences between the amounts of either neutral detergent fiber (NDF) or acid detergent fiber (ADF) incubated and those remaining non-degraded were considered either as degraded NDF or degraded ADF, respectively. Cellulose and hemicellulose contents were calculated based on the differences between NDF and ADL, and NDF and ADF, respectively. The fiber fractions were exclusively expressed in the residual ash.

### Rumen fermentation characteristics and protozoal count

Rumen pH was promptly measured within 2–3 min of sampling after 24 h using a portable pH meter. A 2 mL aliquot of rumen fluid was mixed with an equal amount of methyl-green-formalin-saline solution and stored in a glass bottle at room temperature for microscopic determination of protozoal count and differentiation. This was done using a Digital Zoom Video microscope (LCD 3D, GiPPON; Japan) following the procedure outlined by Dehority et al. (1983).

The contents of the bottles were centrifuged at 10,000 g for 15 min, and the supernatant was utilized to measure the individual SCFAs and ammonia (NH_3_-N) concentrations. The concentrations of SCFAs were measured following the method outlined by De Baere et al. (2013) using reagents and solvents from (Sigma Chemie GmbH, Steinheim, Germany), by High-performance liquid chromatography (HPLC, Agilent 1260 Infinity II with Quaternary Pump, Agilent Technologies, Inc., Santa Clara, California, USA). The detector employed was an Agilent 1260 Infinity Multiple Wavelength Detector (MWD) equipped with a 1024-element photodiode array. The HPLC system included an autosampler (model: 1260 Infinity II Vialsampler) and a separation column (Eclipse XDB-C18, 4.6 × 150, 3.5u Rapid Resol; Agilent Technologies, Inc., Santa Clara, California, USA). A mixture of known concentrations of SCFAs served as an external standard to calibrate the integrator. The mobile phase A consisted of 10-mM KH2PO4 (pH = 2.4 with phosphoric acid), and mobile phase B was Acetonitrile.

Ruminal NH_3_-N concentration was determined calorimetrically in another portion of the supernatant using a spectrophotometer (Alpha-1101 model; Labnics Equipment; California; USA) at a wavelength of 635 nm, employing a commercial lab kit (Biodiagnostic Inc., Giza, Egypt).

### Statistical analysis

The experimental unit was the GP incubation bottle, resulting in 6 static repetitions for each treatment. All data were analyzed using one-way ANOVA via the MIXED procedure of SAS (SAS Institute Inc., Cary, USA, version 9.0). The experimental parameters were treated as a completely randomized design with repeated measures. Statistical significance was determined at *P* ≤ 0.05, and trends were considered at *P* ≤ 0.10.

## Results

### Enzymatic activity of FFNLYFC1 and FFNLYFC2 yeast filtrates

One g of FFNLYFC1 filtrate liberated 102µmol xylose/min from xylan, and 85 µmol glucose/min from CMC, while FFNLYFC2 filtrate liberated 207µmol xylose/min from xylan, and 92 µmol glucose/min from CMC.

### Gas chromatography/mass spectrometry for FFNLYFC1 and FFNLYFC2 yeast filtrates

The GC–MS analysis of filtrates of yeast isolates identified various compounds having different functional groups. 17 phytochemicals were detected in FFNLYFC1 filtrate (Table 3), components belonging to the diisooctyl phthalate,3 isobutylhexahydropyrrolo[1,2-A]pyrazine-1,4-dione, and pyrrolo[1,2-A]pyrazine-1,4-dione, hexahydro-3-(2-methylpropyl) didemnin B) family were most abundant in FFNLYFC1. Results in Table 4 showed that there were 22 phytochemicals detected in FFNLYFC2 where diisooctyl phthalate, ethyl iso-allocholate and anethole were the most abundant components.

**Table 3:** Chemical constituents identified through gas chromatography and mass spectrometry analysis of FFNLYFC1 filtrate

Peaks	Compounds	Retention time (min)	Peak area (%)	Molecular formula
1	Hi-Oleic Safflower Oil	4.86	3.81	C_21_H_22_O_11_
2	Cyclohexanecarboxylic Acid, 2-Hydroxy-, Ethyl Ester	8.27	2.01	C_9_H_16_O_3_
3	Estragole	9.49	6.54	C_10_H1_2O_
4	4-[3-(4-Butoxyphenoxy)Prop Yl]Morpholine	11.45	1.36	C_17_H_27_NO_3_
5	Demycarosylturimycin H	13.84	2.46	C_43_H_74_N_2_O_14_
6	Azacyclododecan-2-One	13.99	3.21	C_11_H_21_N_O_
7	1-Tetradecanol	16.35	2.43	C_14_H_30_O
8	Uric Acid	21.84	3.62	C_5_H_4_N_4_O_3_
9	1,4-Diaza-2,5-Dioxobicyclo[4.3.0]Nonane	22.91	8.06	C_7_H_10_N_2_O_2_
10	Pyrrolo[1,2-A]Pyrazine-1,4-Dione, Hexahydro-3-(2-Methylpropyl)- Didemnin B)	23.88	10.14	C_11_H_18_N_2_O_2_
11	3-Isobutylhexahydropyrro Lo[1,2-A]Pyrazine-1,4-Dione #	25.82	14.4	C_11_H_18_N_2_O_2_
12	Hexadecanoic Acid, Ethyl Ester	27.56	1.34	C_18_H36O_2_
13	Palmitic Acid, TMS Derivative	28.55	1.54	C_19_H_40_O2_Si_
14	6-Octadecenoic Acid	29.52	1.23	C_18_H_34_O_2_
15	9-Octadecenoic Acid (Z-	30.72	1.01	C_18_H_34_O_2_
16	Diisooctyl Phthalate	36.69	28.19	C_24_H_38_O_4_
17	4 H-1-Benzopyran-4-One, 2-(3,4-Dihydroxyphenyl)-6,8-Di-Á-D-Glucopyranosyl-5,7-Dihydroxy-	39.84	2.33	C_27_H_30_O_16_

**Table 4:** Chemical constituents identified through gas chromatography and mass spectrometry analysis of FFNLYFC2 yeast filtrate

Peaks	Compounds	Retention time (min)	Peak area (%)	Molecular formula
1	Ethyl Iso-Allocholate	4.02	1.24	C_26_H_44_O_5_
2	Anethole	9.56	10.04	C_10_H_12_O
3	Bis(Trimethylsilyl)-Loraze Pam	12.12	1.51	C_21_H_26_C_l2_N_2_O_2_Si_2_
4	Cycloheptasiloxane, Tetradecamethyl-	16.29	1.82	C_14_H_42_O_7_Si_7_
5	1 H-Cycloprop[E]Azulen-7-Ol, Decahydro-1,1,7-Trimethyl-4-Methyle Ne-, [1ar-(1aà,4aà,7á,7aá,7bà -	18.77	2.20	C_15_H_24_O
6	Sarreroside	20.08	2.20	C_30_H_42_O_10_
7	Bistrimethylsilyl N-Acetyl Eicosasphinga-4,11-Dienine4H-1-Benzopyran-4-One,	23.40	1.82	C_28_H_57_NO_3_Si_2_
8	2-(3,4-Dimethoxyphenyl)-3,5- Dihydroxy-7-Methoxy-	26.35	4.56	C_18_H_16_O_7_
9	Hexadecanoic Acid, Ethyl Ester	27.60	3.35	C_18_H_36_O_2_
10	2-(3,4,5-Trimethoxyphenyl)- 4 H-3,1-Benzoxazin-4-One	28.58	3.31	C_17_H_15_NO_5_
11	HEXADECANOIC ACID, 2,3-DIHYDROXYPROPYL ESTER	28.71	1.96	C_19_H_38_O_4_
12	Bistrimethylsilyl N-Acetyl Eicosasphinga-4,11-Dienine	29.06	1.26	C_28_H_57_NO_3_Si_2_
13	Oleic Acid	29.54	3.58	C_18_H_34_O_2_
14	9,12,15-Octadecatrienoic Acid, 2,3-Dihydroxypropyl Ester, (Z, Z,Z)	30.61	1.12	C_21_H_36_O_4_
15	Ethyl Oleate	30.74	3.52	C_20_H_38_O_2_
16	Oxiraneoctanoic Acid, 3-Octyl-, Cis- Propanoic Acid	31.24	1.50	C_18_H_34_O_3_
17	2-(3-Acetoxy-4,4,14-Trimethylandrost − 8-En-17-Yl)-	31.52	1.70	C_27_H_42_O_4_
18	2-Hydroxy-3-[(9e)-9-Octadec Enoyloxy]Propyl (9E)-9-Octadecenoate #	32.19	2.40	C_39_H_72_O_5_
19	4 H-1-Benzopyran-4-One, 2-(3,4-Dimethoxyphenyl)-3,5Dihydroxy-7-Methoxy-	33.81	1.46	C_18_H_16_O_7_
20	Ethyl Iso-Allocholate	35.14	11.92	C_26_H_44_O_5_
22	Diisooctyl Phthalate	36.69	29.53	C_24_H_38_O_4_

### Total gas, CH4 and CO2 productions

Table 5 presented the results of supplementation effects of *S. cerevisia* and *P. manshurica* yeast on GP, CH_4_, and CO_2_. The results indicated that no differences were detected among the experimental treatments on the GP values. Among the experimental treatments, the control diet supplemented with AFB1 had the highest (*P* < 0.05) CH_4_ related to OM and TDOM, while FFNLYFC2 and the Mix presented the lowest (*P* < 0.05) values. Mix of *P. manshurica* isolates reduced (*P* < 0.05) CH_4_ related to the fiber fractions compared to control diet supplemented with AFB1 diet. The mix of FFNLYFC1 and FFNLYFC2 was more efficient than the *S. cerevisiae* and the individual yeasts. The Mix also was more efficient in reducing CO_2_ compared to the individual yeasts. The treatment with AFB1 resulted in significant increases (*P* < 0.05) in CO_2_ compared to the control without AFB1 contamination.

**Table 5:** Supplementation effects of *S. Cerevisia* and *P. Manshurica* yeast isolates (FFNLYFC1 and FFNLYFC2) on ruminal gas production (GP), methane emission, and carbon dioxide production

	Treatments						SEM	*P* value
	Control		Yeasts					
			S. cerevisiae	*P. manshurica*				
	-AFB1	+AFB1		FFNLYFC_1_	FFNLYFC_2_	Mix*		
Gas production (ml/ g DM)	153	160	154	156	157	159	12.02	0.999
Methane (CH_4_)								
CH_4_ (ml/g OM)	5.887^ab^	6.539^a^	5.510^ab^	5.22^ab^	4.75^b^	4.911^b^	0.320	0.017
CH_4_ (ml/g TDOM)	8.543^ab^	9.187^a^	7.77^ab^	7.74^ab^	7.27^b^	7.020^b^	0.366	0.011
CH_4_ (ml/g NDF)	19.55^a^	17.05^b^	14.6^c^	17.05^b^	18.23^ab^	13.70^c^	0.451	< 0.0001
CH_4_ (ml/g ADF)	36.96^a^	26.3^b^	25.8^c^	26.7^b^	32.5^ab^	23.93^d^	0.9074	0.003
Carbone dioxide (CO_2_)								
CO_2_ (ml/g OM)	22.2^b^	38.57^a^	38.7^a^	42.2^a^	39.85^a^	23.0^b^	1.995	< 0.0001
CO_2_ (ml/g TDOM)	32.2^b^	58.99^a^	54.6^a^	62.6^a^	56.9^a^	32.99^b^	2.412	< 0.0001
CO_2_ (ml/g NDF)	65.45^c^	147.6^a^	102.9^b^	137^a^	111^b^	66.39^c^	3.041	< 0.0001
CO_2_ (ml/g ADF)	112^c^	263^a^	181^bc^	189^ab^	194^ab^	112^c^	15.67	0.0002

Mix = FFNLYFC1 mixed with FFNLYFC2 in a ratio of 1:1

DM = dry matter, OM = organic matter, TDOM = truly degraded organic matter, NDF = neutral detergent fiber, ADF = acid detergent fiber

SEM = standard error of the mean

^a, b,c^ Means within a row without a common superscript letter differ significantly at *P* < 0.05

### Ruminal nutrient degradability, fermentation parameters and protozoal counts

Results in Table 6 presented the supplementation effects of exogenous *S. cerevisia* and indigenous *Pichia manshurica* yeast on ruminal nutrient degradability. These results showed that the Mix of our endogenous yeasts and the FFNLYFC2 had the highest (*P* < 0.05) OM and fiber fraction degradability compared to the AFB1 diet.

**Table 6:** Supplementation effects of *S. Cerevisia* and *P. Manshurica* yeast isolates (FFNLYFC1 and FFNLYFC2) on ruminal nutrient degradability

	Treatments						SEM	*P* value
	Control		Yeasts					
			*S. cerevisiae*	*P. manshurica*				
	-AFB1	+AFB1		FFNLYFC_1_	FFNLYFC_2_	Mix*		
Nutrient degradability (g/kg DM)								
TDOM	691^a^	653^b^	705^a^	674^ab^	704^a^	710^a^	7.926	0.001
DNDF	343^ab^	261^ab^	372^a^	30^ab^	359^a^	383^a^	17.64	0.003
DADF	192^bc^	146^bc^	211^ab^	121^d^	205^ab^	248^a^	11.50	< 0.0001
Degraded cellulose	213^ab^	119^cd^	201^ab^	89.9^d^	176^bc^	257^a^	14.99	< 0.0001
Degraded hemicellulose	470^ab^	358^b^	509^a^	462^ab^	489^a^	534^a^	24.88	0.005

Mix = FFNLYFC1 mixed with FFNLYFC2 in a ratio of 1:1

SEM = standard error of the mean, TDOM = truly degraded organic matter, DNDF = degraded natural detergent fiber, DADF = degraded acid detergent fiber

^a, b,c, d^ Means within a row without a common superscript letter differ significantly at *P* < 0.05

Table 7 shows the supplementation effects of *S. cerevisia* and *P. manshurica* yeast on ruminal fermentation parameters, SCFAs, and protozoal counts. All the experimental yeasts either the exogenous or the Indigenous resulted in increases (*P* < 0.05) in the ruminal pH compared to the + AFB1 diet. The Mix of the indigenous yeasts had superiority for marinating the pH than the individual ones. There is a wealth of literature confirming the valuable effects of *S. cerevisia* on marinating the pH, but our study is the first one to confirm this effect for the indigenous isolated *P. manshurica* yeast. All the experimental treatments enhanced (*P* < 0.05) the ammonia production compared to the + AFB1 diet. Increases (*P* < 0.05) in the total protozoal count and the *Entodinium spp.* was observed in the P. manshurica diets compared to + AFB1 diet.

**Table 7:** Supplementation effects of *S. Cerevisia* and *P. Manshurica* yeasts (FFNLYFC1 and FFNLYFC2 isolates) on ruminal fermentation parameters, short-chain fatty acids (SCFAs), and protozoal counts

	Treatments						SEM	*P* value
	Control		Yeasts					
				*P. manshurica*				
	-AFB1	+AFB1	*S. cerevisiae*	FFNLYFC_1_	FFNLYFC_2_	Mix*		
Ruminal pH	6.106^bc^	6.036^c^	6.183^ab^	6.17^ab^	6.233^a^	6.156^ab^	0.02494	0.002
Ammonia (mg/100 ml)	28.13^a^	17.88^b^	27.6^a^	26.4^a^	27.04^a^	26.7^a^	1.0389	0.0001
Protozoa (10^5^/ml)								
Entodinium	2.85^c^	3.00^c^	5.10^bc^	9.30^ab^	8.90^ab^	11.9^a^	1.0122	0.0001
Diplodinium	0.600	0.150	0.300	0.200	0.100	0.500	0.1500	0.249
Epidinium	0.300	0.15	0.150	0.200	0.200	0.6000	0.1040	0.070
Isotrica	0.300	0.300	0.1500	0.300	0.300	0.200	0.0540	0.257
Ophryscolex	0.300^ab^	0.450^ab^	1.050^a^	0.200^ab^	0.100^b^	0.100^b^	0.193	0.036
Total	4.35^c^	4.050^c^	6.75^c^	10.20^ab^	9.60^abc^	13.3^a^	1.1812	0.0008
SCFAs (mM)								
Total	118^a^	113^ab^	115^ab^	107^b^	107^b^	121^a^	1.854	0.0007
Acetate	79.07^bc^	74.6^c^	83.24^ab^	76.1^c^	75.23^c^	86.97^a^	1.369	0.0002
Propionate	19.09^b^	19.87^ab^	19.37^ab^	18.8^b^	18.83^b^	21.148^a^	0.4267	0.0152
Butyrate	11.43^a^	11.069^a^	10.76^ab^	9.576^b^	11.187^a^	11.15^a^	0.2773	0.006
Isobutyrate	1.544^a^	0.935^b^	0.955^b^	0.834^c^	0.716^d^	0.614^e^	1.1054	< 0.0001
Valerate	6.32^a^	5.26^ab^	0.552^b^	0.438^b^	0.392^b^	0.478^b^	0.0076	0.004
Isovalerate	1.330^ab^	1.37^a^	1.233^ab^	1.209^abc^	1.014^c^	1.131^bc^	0.044	0.001

Mix = FFNLYFC1 mixed with FFNLYFC2 in a ratio of 1:1

SEM = standard error of the mean

a, b,c, d Means within a row without a common superscript letter differ significantly at *P* < 0.05

Currently, the Mix also enhanced (*P* < 0.05) the total SCFA production which indicated the general enhancements in the fermentation pathways in the expanse of CH_4_ production. The increases in the total SCFAs can be related to the increases (*P* < 0.05) in acetate production, the main contributor to the SCFAs produced by ruminal fermentation.

## Discussion

A diverse array of chemical compounds exhibiting significant biological activity has been identified in the filtrates of FFNLYFC1 and FFNLYFC2. The identified compounds encompass various classes of biological active components. Notably, diisooctyl phthalate that known with antimicrobial activity against wide broad-spectrum of pathogenic bacteria (Ngidiet al. 2021) emerged as the predominant constituent in both FFNLYFC1 and FFNLYFC2 filtrates. This result suggests that both *P. manshurica* isolates have antibacterial activity. However, distinct compositions were observed between FFNLYFC1 and FFNLYFC2, with isobutylhexahydropyrrolo[1,2-a]pyrazine-1,4-dione comprising 14.4% in FFNLYFC1, while ethyl iso-allocholate and anethole were dominating at 11.92% and 10.24% in FFNLYFC2, respectively.

The principal active components, including isobutyl hexahydropyrrolo[1,2-a]pyrazine-1,4-dione detected in FFNLYFC1is recognized for its substantial biological effects. Previous studies have documented the antifungal and anti-inflammatory properties of isobutylhexahydropyrrolo[1,2-a]pyrazine-1,4-dione (Xu et al. 2009). Moreover, recent investigations have implicated it as a potent antimicrobial metabolite involved in bacterial defense mechanisms (Mirzaee et al. 2021). Ethyl iso-allocholate and anethole that found in FFNLYFC2 are acknowledged for their antimicrobial, antioxidant, and anticancer activities (El Mihyaoui et al. 2023). The observed disparities in metabolite profiles between the two yeast isolates, despite their shared media and yeast species, suggest potential differences between FFNLYFC1 and FFNLYFC2 in their effects on ruminal fermentation and nutrient degradability, thus we determined FFNLYFC1 and FFNLYFC2 and their mixture in a ratio of 1:1 in an in vitro assay.

The results of the GP experiment indicated no differences in GP values observed among the experimental treatments. These results were somewhat unexpected since the literature reported that AFB1 (at concentrations of 5 and 10 µg/ml ruminal inocula) adversely affected the GP values compared to the control diet without AFB1 (Khodabandehloo et al. 2019; Soltan et al. 2022). Similarly, Mojtahedi et al. (2013) found that the addition levels of 0 to 900 AFB1 ng/ml reduced GP values from 196 to 166 ml/g DM, respectively. In the current study, the adverse effects of AFB1 on GP were not observed. Explanation for this result are not clear, however the difference in the diet substrate can affect the obtained results.

Among the experimental treatments, the mix of *P. manshurica* isolates was more efficient in reducing CH_4_ values expressed in relation to the fiber degradability (ADF) than the *S. cerevisiae* and the individual *P. manshurica* isolates. We detected cellulase and xylanase activities in both FFNLYFC1 and FFNLYFC2 isolates, thus it seems that these yeasts are efficient to degrade fiber in the AFB1-contaminated diets under ruminal degradation. It is worth noticing that the source of the ruminal inocula came from calves of the in vitro incubation, thus it seems that these yeasts can affect a wide board of ruminal fermentation regardless of the animal type. The mix of these yeasts seems to be more favorable than the individual ones in cellulose degradation. These results indicated that these both FFNLYFC1 and FFNLYFC2 have a synergistic effect concerning fiber degradability. The increases in OM, NDF, ADF cellulose and hemicelluloses by the Mix treatment compared to the control with AFB1 contamination may confirm its ability for fiber degradation. This also suggests that the decrease in CH_4_ levels caused by mix of *P. manshurica* was not attributed to microbial fermentation inhibition, but could be attributed to a potential influence on the methanogenesis process (Malik et al. 2022). However, this potential has not yet been substantiated; the identification of unique biological active components of both *P. manshurica* isolates may support this hypothesis. There are no evidences supporting the direct efficacy of incorporating *s. cerevisiae* for the adsorption of AFB1 or releasing cellulase or xyalnsae fiber degrading enzymes (Mirzaei et al. 2020), however our results indicated positive effects of *S. cerevisiae* on ruminal fiber degradability of AFB1 contaminated diet. These findings suggest that *S. cerevisiae* may also possess the potential to alleviate the adverse effects of AFB1 on nutrient degradability in somehow. Recently, Mirzaei et al. (2020) found reduction in concentration of aflatoxin M1 (the metabolite of AFB1) in both serum and milk by incorporating *S. cerevisiae* in diets of the transition cows through either enhancing the cow helath and immunity status (Razavi et al. 2019) or supporting the nutrient degradability by providing an ecology suitable for the function of ruminal cellulite bacteria.

All the AFB1 diets either with or without yeasts supplementations had higher CO_2_ production than the control diet without ABB1 contamination, this except for the Mix of *P. manshurica* treatment. These results indicated that Mix of *P. manshurica* was the most promising treatment in reducing the GHG from diets contaminated with AFB1. All the experimental yeasts either the exogenous or the indigenous resulted in increases in the ruminal pH compared to the AFB1 control diet. These conditions are favorable for the dairy animals to avoid the risk of acidosis (Soltan et al. 2021a). The Mix of the indigenous yeasts had superiority for marinating the pH than the individual ones. There is a wealth of literature confirming the valuable effects of *S. cerevisia* on marinating the pH (Sousa et al. 2018), but our study is the first one to confirm this effect for the indigenous isolated *P. manshurica* yeast.

The impact of the *S. cerevisia*, and *P. manshurica* diets on ruminal pH can be linked to NH_3_-N production (Guo et al. 2022), since all the experimental treatments enhanced the NH_3_-N production compared to the AFB1 diet, these results indicated that these yeasts may affect the protein metabolism or enhance the microbial protein synthesis (Soltan et al. 2021b). The increases in the total protozoal count especially the *Entodinium spp.* may confirm such hypothesis. It seems that somehow the Mix of the experimental yeasts contributed indirectly to the fiber degradation through enhancements in the protozoa populations as *Entodinium spp.* are the highest number of protozoal species found in the normal rumen conditions (Park et al. 2019). This may partly explain the high OM and fiber degradability caused by Mix of *P. manshurica* as some studies have suggested that *Entodinium species* may be more abundant and diverse in rumen environments and may contribute more significantly to fiber degradation compared to other protozoal species (Park et al. 2019). It seems that somehow the Mix of the experimental yeasts contributed indirectly to the fiber degradation through enhancements in the protozoa populations. Protozoa can indirectly increase ruminal CH_4_ production, as the studies have established a synergistic relationship between protozoa and methanogens, protozoa produce end metabolites such as H₂ that support methanogen activity. Therefore, measuring protozoal counts can help determine whether treatments have a direct or indirect impact on CH₄ emissions (Soltan et al. 2021c). The enhancement of total protozoa by the Mix of *P. manshurica* may indicate that the reduction in CH₄ was a result of direct antibacterial activity affecting methanogenesis, as both protozoal counts and fiber degradation were enhanced.

Unlike FFNLYFC1 or FFNLYFC2, their Mix shifted the profile of SCFA production towards acetate rather than propionate. The reasons for these results are not clear; nevertheless, it could be suggested that the unique combination of FFNLYFC1 and FFNLYFC2 active components might influence ruminal microbes through various potent mechanisms of action, such as antioxidant and antimicrobial properties, potentially leading to enhance the ruminal pH and consequently the acetate proportion (Russell and Strobel 1989; Soltan et al. 2021b). Enhancements in ruminal pH may also explain the increases in total SCFA concentration and total protozoa populations by the Mix of *P. manshurica* treatment compared to its individual isolates. It is known that most acetate producers and protozoal populations in the rumen are sensitive to acidic conditions, whereas acid-tolerant species are propionate producers (Patra et al. 2023). Therefore, the increases in pH resulting from Mix administration may lead to a shift in SCFA production, favoring a higher acetate formation when compared to the individual *P. manshurica* isolates treatment. The increases in ruminal pH can affect the protein solubility and reduce the concentrations of branched-chain volatile fatty acids (Patra et al. 2023). This partially explains the decrease in isovalerate and isobutyrate proportions due to the Mix treatment.

It seems that the utilization of the Mix resulted in the inhibition of CH_4_ formation by promoting SCFA and acetate productions. Formation of SCFA competes with CH_4_ production for available H + and CO_2_ within the rumen, ultimately leading to decreased GHG emissions (Patra et al. 2023). Generally, the enhancements in SCFA production caused by either individual isolates of *P. manshurica* or their mixture can be a result of the average of all the fermentation processes happening in the rumen, thus any increases in the SCFA can save the animal growth energy and enhance the conversion into milk or meat.

## Conclusions

The filtrate of both *P. manshurica* isolates exhibited a notable presence of biologically active phytochemical antimicrobial metabolites, including diisooctyl phthalate. The occurrence of AFB1 contamination within ruminant diets was correlate with increased ruminal CH_4_ production, that linked to alterations in fiber degradability, pH levels, populations of *Entodinium spp.*, and total protozoal counts. The addition *P. manshurica* isolates mixture demonstrated promising efficacy in reducing GHG emissions within rumen environments in AFB1 contaminated diets, this supplementation exhibited superior effects compared to individual isolates of *P. manshurica* or *S.cerevisiae* concerning rumen fermentation, pH regulation, abundance of *Entodinium spp.* and total protozoa, as well as the production of SCFA and acetate. These findings can be attributed to numerous factors, including the specific animal species under study, the formulation of the *P. manshurica* supplement, and environmental conditions that influence mycotoxin proliferation and animal metabolism. Thus, future investigations into the supplementation of the *P.manshurica* isolates mixture should prioritize the comprehensive examination of qualitative and quantitative characteristics of ruminal microorganisms involved in methanogenesis and fermentation processes in long-terms in vivo studies.

## Data Availability

All data of this manuscript are included in this article version.
